# PD-1 expression contributes to functional impairment of NK cells in patients with B-CLL

**DOI:** 10.1038/s41375-024-02271-1

**Published:** 2024-05-09

**Authors:** Mustafa Farhat, Wayne Croft, Helen M. Parry, Kriti Verma, Francesca A. M. Kinsella, Jinsong Xu, David Bone, Tina McSkeane, Shankara Paneesha, Guy Pratt, Paul Moss, Jianmin Zuo

**Affiliations:** 1 Institute of Immunology and Immunotherapy, College of Medical and Dental Sciences, University of Birmingham, Birmingham, B15 2TT UK; 2 University Hospitals Birmingham NHS Foundation Trust, Queen Elizabeth Hospital Birmingham, Mindelsohn Way, Edgbaston, Birmingham, B15 2GW UK; 3 Birmingham Heartlands Hospital, University Hospitals Birmingham, Birmingham, B9 5SS UK

**Keywords:** Tumour immunology, Translational research

B cell chronic lymphocytic leukemia (B-CLL) is a common leukemia subtype [1] and compromised immune function is a major clinical problem leading to increased mortality among patients [2].

Natural killer (NK) cells recognize and kill transformed or virally infected cells through mechanisms including granule-mediated cell lysis, cytokine release, and antibody-dependent cell-mediated cytotoxicity (ADCC) [3, 4]. However, NK cells are frequently dysfunctional in patients with cancer, and this profile is also seen in patients with CLL. An imbalance in the relative expression of activating and inhibitory receptors is an important mechanism of immune evasion, with decreased expression of the dominant activatory receptors NKG2D, DNAM-1 and NCRs as a common feature. This phenotype is associated with reduced degranulation and impaired lysis of target cells [5–7].

Recent studies have shown that NK cells can express a range of inhibitory checkpoint proteins that may also negatively regulate the activity of NK cells. In particular, PD-1 expression has been demonstrated in patients with several cancer subtypes, including hematological malignancies [8, 9], and is associated with functional impairment which can be reversed by PD-1 blockade [10, 11].

It is well established that engagement of PD-1 on T cells with its PD-L1 ligand can inhibit T cell functional responses to CLL tumor cells and mediate an exhausted T cell phenotype [12, 13]. Indeed, PD-1 blockade in patients with B-CLL, either as a single agent therapy or in combination with ibrutinib, has elicited clinical responses [14], although the role of PD-1 on NK cells in patients with B-CLL has not been explored.

Here we investigated the expression of a range of checkpoint receptors, including PD-1, on NK cells from B-CLL patients. We characterized the phenotype and functional capacity of PD-1^pos^ NK cells compared to their PD-1^neg^ counterparts and evaluated the impact of PD-1:PD-L1 blockade on the tumor-specific activity of NK cells against both primary NK cells and NK lines.

Initial studies used flow cytometry to determine the expression of checkpoint receptors on NK cells from 72 untreated stage A CLL patients and age-matched healthy controls (HDs) (Clinical demographic information in Supplementary Table). As such, the single or combined expression of PD1, TIGIT, CTLA-4, LAG3, CD96, TIM-3, TIGIT and NKG2A was defined on CD3-CD56 + NK cells within peripheral blood (Gating strategy shown in Supplementary Fig. 1). A 2.2-fold increase in the NK cell count was seen in B-CLL patients compared with HDs (407 ± 53 /µl vs. 183 ± 74 respectively) (Supplementary Fig. 2A). The expression of multiple checkpoint proteins was increased on NK cells from patients. Of note, the median percentage of PD-1 + NK cells was more than doubled in patients at 0.8% compared to 0.3% in HDs (*p* < 0.001), while this subset comprised up to 22% of NK cells in some donors (Fig. 1A). The median expression of CTLA-4 and CD96 was also seven-fold and nine-fold higher on NK cells from patients (CTLA-4 + 2.3% vs. 0.3%; p < 0.0001: CD96 + 4.8% vs. 0.5%; *p* < 0.0001). (Fig. 1A). CTLA-4 expression did not display a relative increase on PD-1 + NK cells indicating an independent expression pattern (Supplementary Fig. 2).

**Fig. 1:**
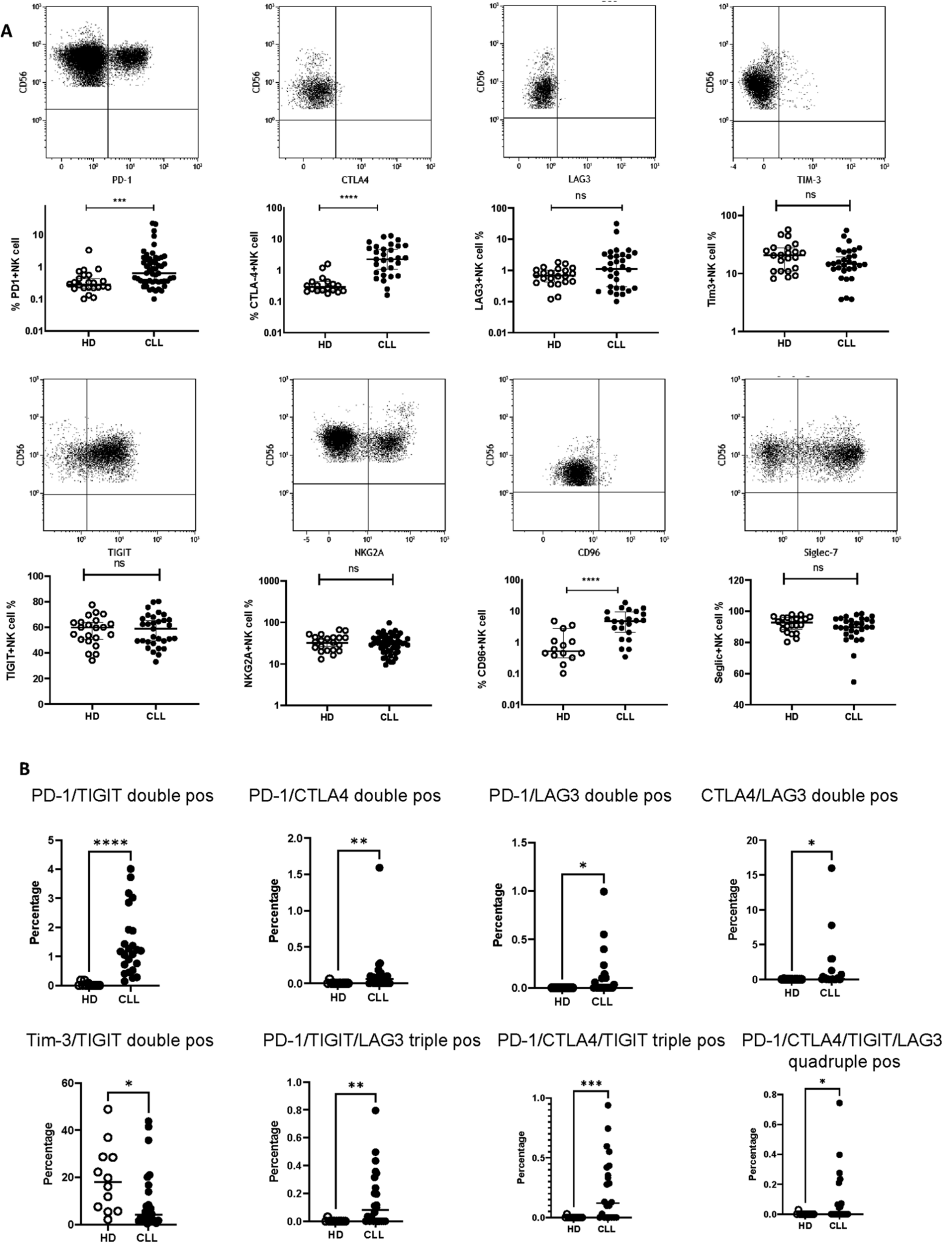
Expression pattern of immune checkpoint receptors on NK cells from B-CLL patients. **A** Surface expression of PD-1, CTLA-4), LAG-3, TIM-3), TIGIT, NKG2A, CD96 and Siglec-7 on NK cells from CLL patients was compared to age-matched healthy controls (HD). Flow plots to demonstrate representative data from one B-CLL patient and dot graphs are the comparisons between B-CLL patients and HD. **B** Coexpression of PD-1, CTLA-4, LAG-3, TIM-3 and TIGIT on NK cells was analyzed using Boolean gating. Percentages were compared between B-CLL and age-matched healthy controls (HD) for double, triple and four and five checkpoint receptor coexpressed NK cell populations. Statistical analysis was performed using the Mann-Whitney nonparametric test (*p < 0.05, **p < 0.01, ***p < 0.001 and ****p < 0.0001).

The immune checkpoint co-expression pattern of PD-1, CTLA-4, LAG-3, TIGIT and TIM-3 was further examined using Boolean gating. The expression of a range of checkpoint combinations was increased in the patient group (Fig. 1B). In particular, the proportions of PD-1/TIGIT; CTLA4/LAG3; PD-1/CTLA4; PD-1/LAG3; PD-1/TIGIT/LAG3; PD-1/CTLA4/TIGIT; and PD-1/CTLA4/TIGIT/LAG3 co-expressing NK cells were all increased in the patient group. Co-expression of the strong inhibitory checkpoints PD-1 and TIGIT was noteworthy as a phenotype not observed in healthy donors but comprising over 2% of the NK cell pool in many patients. Furthermore, it is notable that PD-1 expression is a component in 6 of the 7 checkpoint combinations that are increased on NK cells in patients, implying a potentially important central role for PD-1 in the checkpoint regulation of NK cells in CLL.

Given the importance of PD-1 in combinatorial checkpoint expression we next focused our studies on the phenotypic and functional features of PD-1 + NK cells (PD-1^pos^). The majority of PD-1^pos^ NK cells were present within the cytotoxic CD56^dim^ subset, the dominant population within blood and a more differentiated pool compared to CD56^bri^ NK cells. In particular, within a subset of 10 patients where PD-1^pos^ cells comprised 8.9% of the total NK pool, PD-1 was expressed on 9.1% of the CD56^dim^ population compared to only 0.76% of CD56^bri^ cells (Fig. 2A).

**Fig. 2:**
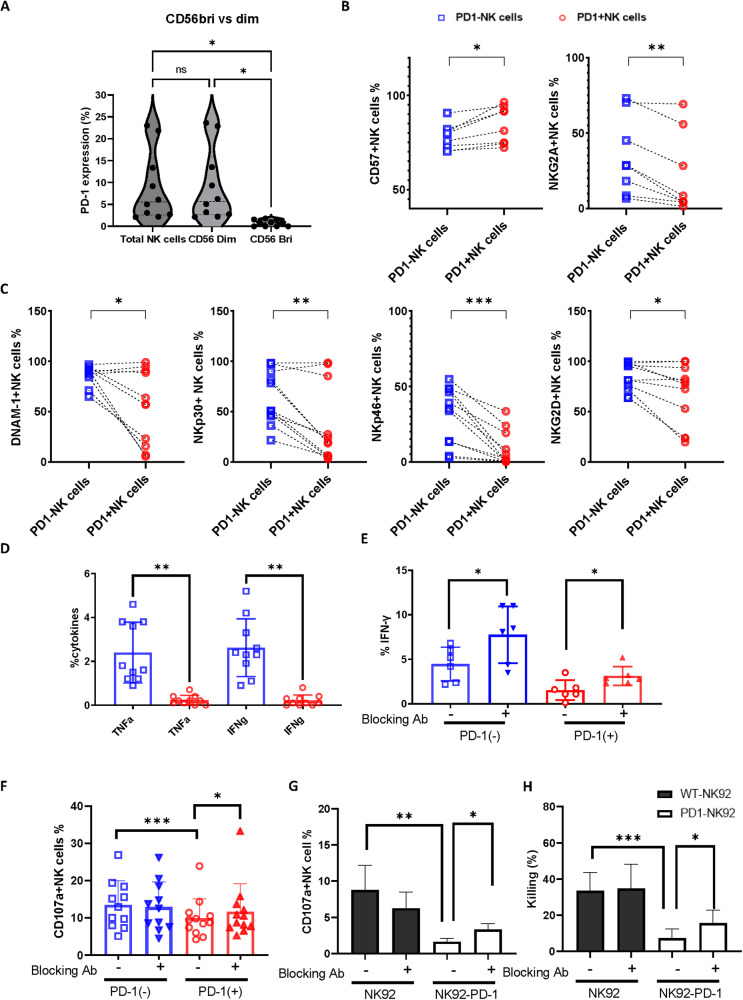
PD-1 NK cells express lower levels of activating receptors and reduced functionality, which can be partially reversed. **A** The percentages of PD-1^pos^ NK cells were compared between the CD56^bri^ and CD56^dim^ NK populations. **B** Dot plots to compare the percentages of CD57^pos^ and NKG2A^pos^ NK cells between PD-1^pos^ NK cells (red circles) and PD-1^neg^ NK cells (blue squares). **C** Dot plots to compare the percentages of DNAM-1^pos,^ NKp30^pos^, NKp46^pos^, and NKG2D^pos^ NK cells between PD-1^pos^ NK cells (red circles) and PD-1^neg^ NK cells (blue squares). Statistical significance was determined by Wilcoxon matched-paired nonparametric test using GraphPad Prism (*p < 0.05, **p < 0.01, ***p < 0.001). **D** Bar charts summarizing IFN-γ and TNF-α production by PD-1^pos^ (red circles) vs. PD-1^neg^ (blue squares) NK cells from B-CLL patients after stimulation with 721.221 (n = 10). **E** Bar charts summarizing IFN-γ production by PD-1^pos^ NK cells (red) and PD-1^neg^ NK cells (blue) before and after PDL-1/L-2 blockade. Each dot represents a single donor. The differences were determined by the Mann-Whitney test using GraphPad Prism (*p < 0.05, **p < 0.01). **F** Bar chart to compare CD107a expression after stimulation with 721.221 by PD-1^pos^ NK cells (red) and PD-1^neg^ NK cells (blue) before and after PDL-1/L-2 blockade. Data are shown as the percentage of CD107a-expressing NK cell subsets (*n* = 10). **G** Summary of CD107a expression in PD-1^pos^ NK-92 (white bars) and PD-1^neg^ NK-92 (black bars) cells after stimulation with 721.221 before and after PDL-1/L-2 blockade. **H** Bar charts to compare the cytotoxicity capacity of PD-1^pos^ NK-92 (white bars) and PD-1^neg^ NK-92 (black bars) against 721.221 target cells with and without PDL-1/L-2 blockade. Data were analyzed using Mann-Whitney and Wilcoxon matched-paired nonparametric tests (*p < 0.05, **p < 0.01, ***p < 0.001).

The expression of differentiation and activating receptors was next determined on PD-1^pos^ and PD-1^neg^ NK cells. PD-1^pos^ populations expressed high levels of the late differentiation marker CD57 (85 ± 3.5%) but less expression of NKG2A, a protein typically found on less mature NK subsets (22 ± 9.4%). Comparable values were 78 ± 2.4% and 35 ± 9.1% for PD-1^neg^ NK cells respectively (*p* = 0.0078 for both) (Fig. 2B). Importantly, the expression of the NK-activating receptors NKG2D, NCRs and DNAM-1 was substantially downregulated on PD-1^pos^ NK cells compared to PD-1^neg^ populations (Fig. 2C). In particular, NKG2D was expressed on 66% ±9.3% of PD-1^pos^ NK cells compared to 84 ± 4.3% of PD-1^neg^ NK cells (*p* = 0.0137). Downregulation of NKp30 and NKp46 was even more dramatic, with expression values of 36 ± 12% vs. 63 ± 8% (*p* = 0.049), and 8.5 ± 3.5% vs. 31 ± 6%, respectively (*p* = 0.001). DNAM-1 expression was reduced by 62% on PD-1^pos^ populations, present on 33 ± 11% of cells compared to 87 ± 3% of PD-1 ^neg^ cells (*p* = 0.023).

scRNA-Seq was used to compare the transcriptome of FACS-sorted PD-1^pos^ and PD-1^neg^ NK cells but revealed relatively subtle differences between populations (Supplementary Fig. 3). Transcription of *PDCD1*, the gene encoding PD-1, was observed in the PD-1^pos^ subset, indicating that trogocytosis is not the sole determinant of protein expression [15]. Of note, the transcriptomes of PD-1^pos^ and PD-1^neg^ NK cells did not separate into two distinct clusters, implying that PD-1 expression is not a dominant transcriptional NK lineage marker. Enrichment for genes associated with cytotoxicity and immune synapse formation may reflect high levels of ongoing activation.

The functional relevance of PD-1 expression on NK cells in patients with B-CLL was next explored. NK cells were stimulated with PD-L1-positive 721.221 target cell lines that express both PD-L1 and PD-L2 (Supplementary Fig. 4) and cytokine production was measured using intracellular flow cytometry. IFN-γ and TNF-α production was 10-12-fold lower from PD-1^pos^ cells compared to PD-1^neg^ subsets (IFN-γː 0.2 ± 0.08% vs. 2.6 ± 0.4% (*p* = 0.002); TNF-αː 0.2 ± 0.07% vs. 2.4 ± 0.4% (*p* = 0.002)) (Fig. 2D). To assess whether impairment of cytokine production could be reversed by blockade of PD-1 with its ligand, NK cells were stimulated in the presence of antibodies against PD-L1 and PD-L2. Indeed, PD-1:PD-L blockade doubled the proportion of IFN-γ+ PD-1^pos^ NK cells (3.1 ± 0.4% vs. 1.5 ± 0.5%; *p* = 0.0313) (Fig. 2E). Of interest, PD-L blockade had no impact on the relative proportion of TNF-α + NK cells following activation (data not shown), potentially reflecting the impact of additional checkpoint receptors on PD-1^pos^ cells.

We next assessed CD107a expression as a marker of stimulation-induced NK cell degranulation. CD107a expression was observed on only 9.9 ± 1.6% of PD-1^pos^ NK cells compared with 13.5 ± 1.9% of PD-1^neg^ NK cells (*p* = 0.001; Fig. 2F). This value increased to 11.6 ± 2.2% after PD-L blockade (*p* = 0.027) (Fig. 2F) whilst no increase was observed with PD-1^neg^ NK cells.

As the number of PD-1^pos^ NK cells was too low to undertake a direct cytotoxicity assay, the functional impact of PD-1 expression was determined by transduction of the NK-92 cell line using a retroviral vector (Supplementary Fig. 3). Flow cytometry was then used to determine the relative degranulation and cytotoxic ability against the 721.221 cell line. In line with observations from primary cells, PD-1^pos^ NK-92 cells expressed markedly lower CD107a levels than control NK-92 cells following stimulation with 721.221 (1.6 ± 0.2% vs. 8.8% ± 1.5% (*p* = 0.0079)) (Fig. 2G). Cytotoxicity against PD-L1/2-expressing 721.221 cells was also substantially reduced (7.4 ± 1.7% vs. 33 ± 3.4% (*p* < 0.001)) (Fig. 2H). Importantly, antibody blockade against PD-L1 and PD-L2 increased CD107a expression to 3.3 ± 0.4% (*p* = 0.016) (Fig. 2G) whilst cytotoxicity increased to 16 ± 2.4% (*p* = 0.024) (Fig. 2H).

In conclusion, the expression of a range of inhibitory checkpoint proteins is increased on NK cells in patients with CLL and PD-1 is a common shared factor in many cases. PD-1^pos^ cells have impaired functional activity but this may be partially improved by inhibition of ligand engagement. Enhancement of NK cell function may therefore contribute to the clinical utility of antibody mediated PD-1 checkpoint blockade in patients with B-CLL.

### Supplementary information

Supplementary Material

## Data Availability

The newly generated scRNA seq data reported in this paper have been deposited in the Gene Expression Omnibus (https://www.ncbi.nlm.nih.gov/geo/) with the accession number of GSE252613. All raw datasets and the processed datasets are available from the corresponding author upon reasonable request.
