# Supplementary material for: PD-1 expression contributes to functional impairment of NK cells in patients with B-CLL
Source: Leukemia. 2024 May 9;38(8):1813–7. doi: 10.1038/s41375-024-02271-1 (PMC11286510; doi:10.1038/s41375-024-02271-1)
Supplement: Supplementary file 1 — Supplementary Material [file 41375_2024_2271_MOESM1_ESM.pdf]

Materials and methods

Patients and Healthy control cohorts

About 9ml of heparinised whole blood were received from 72 untreated and Binet stage A, B-CLL patients from outpatient haematology clinics at Queen Elizabeth Hospital Birmingham (QEHB), and Heartlands Hospital Birmingham (HHB) following written informed consent form (REC no 10/H1206/58) with the median age of 72±7 years. Age-matched healthy volunteers (HD) were recruited (median age 69+15 years old) with written and informed consent form (REC no 2002/073). 9ml of peripheral blood were collected in heparinised tubes from volunteers by trained phlebotomist. Healthy volunteers were used as controls in this study.

Reagents and antibodies

RPMI-1640 medium and Phosphate Buffered saline (PBS) were purchased from sigma-Aldrich-UK. Pen/Strep and L-glutamine were from Gibco Thermo Fisher-USA. Whilst CFSE was from eBioscience Thermo Fisher-UK, Fetal Bovine albumin, Saponin and Brefeldin A were all from Sigma-Aldrich-USA. Interleukine-2 (IL-2) was from PeproTech-USA.

Florescent labelled anti-human monoclonal antibodies used in flow cytometry analysis of NK cell phenotype were CD56-APC-Cy7, CD56-PE/Cy-7, CD3-AF700, PD-1-PE, CD16-FITC, CTLA-4-PerCP/Cy5.5, LAG-3-PE/Cy7, TIGIT-APC, TIM-3-Pacific blue, Siglic-7-PE, CD96-PE, NKG2A-APC, CD57-Pacific blue, DNAM-1-PE-Dazzle, NKG2D-PE, NKp46-PE-Cy7, NKp30-APC. All these antibodies were purchased from BioLegend except last one was from Miltenyi Biotec. The anti-human monoclonal antibodies used to study the functionality of NK were TNF-a-AF-488, IFN-g-AF-700 and CD107a-FITC and in addition to purified anti human PDL1 and PDL2 which all were purchased from BioLegend-UK. The fixable viability dye 780 was from Thermo Fisher-USA. The expression of these immune checkpoints was determined based on fluorescence minus one control (FMO).

Cell lines

721.221 cancer cell lines were purchased from ATCC. The cells were cultured in RPMI-1640 medium with 10% FBS and 5% of pen/strep and 5% L-glutamine and used as a target cells to study the functionality of NK cells. NK-92 and PD-1^{pos} NK-92 cells were grown with NK-92 media, which is RPMI-1640 medium supplemented with 10% horse serum and 5% human serum and 400IU rhIL-2.

NK cell surface staining

$1x10^6$ PBMCs from fresh or frozen PBMCs of B-CLL patients or HD were washed with 2ml PBS at 413x g for 5 minutes and supernatant was discarded. Cells were resuspended in the residual supernatant

(about 50µl) and co-incubated with the relevant antibodies panel at 4°C for 20 to 25 minutes in dark place. 2ml PBS was then added to each tube and centrifuged at 413x g for 5 minutes. The supernatant was discarded, and cells were resuspended in the residual and topped with 200µl PBS. 1µl of live/dead PI dye was added to each tube just prior sample acquisition on the Gallios™.

Intracellular cytokines staining of NK cells

2 x 10^6 PBMCs from B-CLL or HD were co-cultured with and without K562 target cells at 1:1 ratio for overnight in the presence of Brefeldin A in a total volume of 0.5ml growth media, at 37°C, 5% CO2. Next day, cells were washed with PBS and resuspended in the residual buffer and stained with appropriate surface antibodies panel with fixable viability dye 780 1µl/tube of cell suspension for 30 minutes at 4°C. Cells were then washed with 2ml PBS and supernatant was discarded. Cells were resuspended and gently vortexed in100ul of 4% paraformaldehyde (PFA) for 15 minutes for fixation. Cells were washed with 4ml PBS at 645x g for 10 minutes, resuspended in the residual volume and permeabilised in 100ul of 0.5% Saponin for 5 minutes in the dark at room temperature with gentle mixing. In the presence of saponin, cytokine antibodies panel was added subsequently and incubated for 30 minutes at room temperature in the dark. Cells were then washed with PBS and resuspended in 200µl PBS buffer for flow cytometry acquisition.

Functional assays and PD-1 ligands blockade experiments

Degranulation assay: Fresh or frozen PBMCs from B-CLL patients with high percentage of PD-1^{pos} NK cells were used in this experiment. The degranulation assay was set up using purified NK cells. NK cells were enriched using a negative selection EasySepTM Haman NK cell Enrichment Kit. Purified NK cells were counted, washed and co-culture with 721.221 cells at E:T ration 1:5. After one hour of incubation anti CD107a-FITC antibody was added into all wells with gentle mix to capture the CD107a molecules on the surface of degranulating NK cells. To assess the effect of blocking PD-1 signalling, anti PDL1 and anti PDL2 blocking antibodies was added in the corresponding wells. After 5 hours, cells were washed and stained with NK cell markers and analysed using GalliosTM.

PD-1^{pos} NK cell line cytotoxicity assay

The cytotoxicity of PD-1 transduced NK cell lines (PD-1^{pos} NK-92 cells) were investigated against PDL1/L2 expressing 721.221 target cells and compared with their corresponding PD-1^{neg} NK-92 cell line. The flow cytometry-based killing assay was used to measure the capacity of cytotoxicity of PD-

1^{pos} versus PD-1^{neg} NK-92 cells by quantifying the number of viable CFSE-labelled target cells following overnight co-incubation.

PD-1^{pos} NK-92 and wt-NK-92 cell lines were harvested from culture media and were washed with growth media and resuspended. Meanwhile, 721.221 target cells were harvested and labelled with CFSE. Cells were plated in duplicate using sterile 96 well plate at 5:1 E:T ratio for both NK cell types with 721.221. To assess the effect of blocking PD-1 signalling, anti PDL1 and anti PDL2 blocking antibodies was added in the corresponding wells. Plates were incubated at 37°C in 5% CO2 for overnight (16 hours). The controls were set up with target cells 721.221 only and growth media in duplicate.

Next day, cells were transferred to FACS tubes without wash, and 10μl of vortexed CountBright beads was added to each tube. Tubes were topped up with PBS and PI was added to each tube just prior cell acquisition on Gallios™. All tubes were run on Gallios™ with equal time (2 minutes) at high flow rate. Data was analysed on Kalusa software. Live target cell numbers were calculated according to the counting beads.

scRNA- seq of NK cells

scRNA seq experiment was performed to study the transcription profile of PD-1^{pos} versus PD-1^{neg} NK cells from B-CLL patients. scRNA-seq was achieved using 2 fresh PBMCs form B-CLL patients with high percentage of PD-1^{pos} NK cells (one with 22% and one with 13% PD-1^{pos} NK cells). Initially, NK cells were quick enriched using negative selection EasySepTM Haman NK cell Enrichment Kit as described previously. Enriched NK cells were stained with CD56-PE/Cy7, CD3-FITC, PD1-PE, CD19 and CD14-PB and viability dye (flour506-PB) and FACS sorted to obtain pure NK cells higher than 99%. NK cells were spun down and resuspended in growth media at 1000 cells/ul then sent to Genomics Birmingham-University of Birmingham to check the viability and sequencing . The generated library pool passed the QC in terms of concentration and fragment size as determined using Qubit and TapeStation and was sequenced using a NextSeq 500 (Illumina) platform. The median number of sequenced NK cells returned was 3,500 with 1,500 unique molecular identifiers (UMIs) per cell.

Raw sequencing data was processed using CellRanger version 2.1.1(1) with *mkfastq* and *count* functions to convert to fastq, align to human genome GRCh38 and generate filtered feature count matrices for each sample. Count matrices were imported into R version 3.6.2 as single cell expression (SCE) objects and log normalised expression values calculated after adjusting for size factors using *multiBatchNorm* from the R package *scran(2).* Data from each sample were merged into a single object and QC metrics calculated using *scater* package(3). Genes were filtered to remove those with < 2 reads

and cells were filtered removing those with high mitochondrial related gene expression (>15%). The top 2000 highly variable genes were used with fast mutual nearest neighbour correction to correct for batch effects using *fastMNN(4).* Dimensionality reduction was performed by t-Distributed Stochastic Neighbour Embedding (tSNE) on MNN-corrected data to produce 2D embeddings.

To test for differential expression of genes between $PD1^{pos}$ and $PD1^{neg}$ cells the *scran* function *findMarkers* was used with patient as a blocking factor. Genes were regarded as differentially expressed if false discovery rate (FDR) < 0.01 and absolute log fold change > 0.1

Statistical analysis

Mann-Whitney nonparametric test, Wilcoxon matched-paired non-parametric were used to compare the differences between the groups. All statistical analysis were performed using GrapPad Prism 9 (GraphPad Prism software Inc, version 9.3.1 USA). A difference was considered to be statistically significant when two-sided p test is less than 0.05.

References

1. Zheng GX, Terry JM, Belgrader P, Ryvkin P, Bent ZW, Wilson R, et al. Massively parallel digital transcriptional profiling of single cells. Nat Commun. 2017;8:14049.
2. Lun AT, McCarthy DJ, Marioni JC. A step-by-step workflow for low-level analysis of single-cell RNA-seq data with Bioconductor. F1000Res. 2016;5:2122.
3. McCarthy DJ, Campbell KR, Lun AT, Wills QF. Scater: pre-processing, quality control, normalization and visualization of single-cell RNA-seq data in R. Bioinformatics. 2017;33(8):1179-86.
4. Haghverdi L, Lun ATL, Morgan MD, Marioni JC. Batch effects in single-cell RNA-sequencing data are corrected by matching mutual nearest neighbors. Nat Biotechnol. 2018;36(5):421-7.

**Number of patients**		**72**
**Age at time of study (years)**	**Median**<br>**IQR**<br>**Range**	**72**<br>**65-79**<br>**41-93**
**Sex**	**Men**<br>**Women**	**40/73 (55%)**<br>**33/73 (45%)**
**Ethnicity**	**White Caucasian**<br>**SE Asian**	**72/73**<br>**1/73**
**Time since diagnosis (years)**	**Median**<br>**IQR**	**4.4**<br>**1.98-8.6**
**Never treated**<br>**Watch and wait at time of sampling**<br>**Subsequently treated**		**72/72 9100%)**<br>**72/72 (100%)**<br>**9/73**

Supplementary Table : Demographic summary of the CLL patients cohort

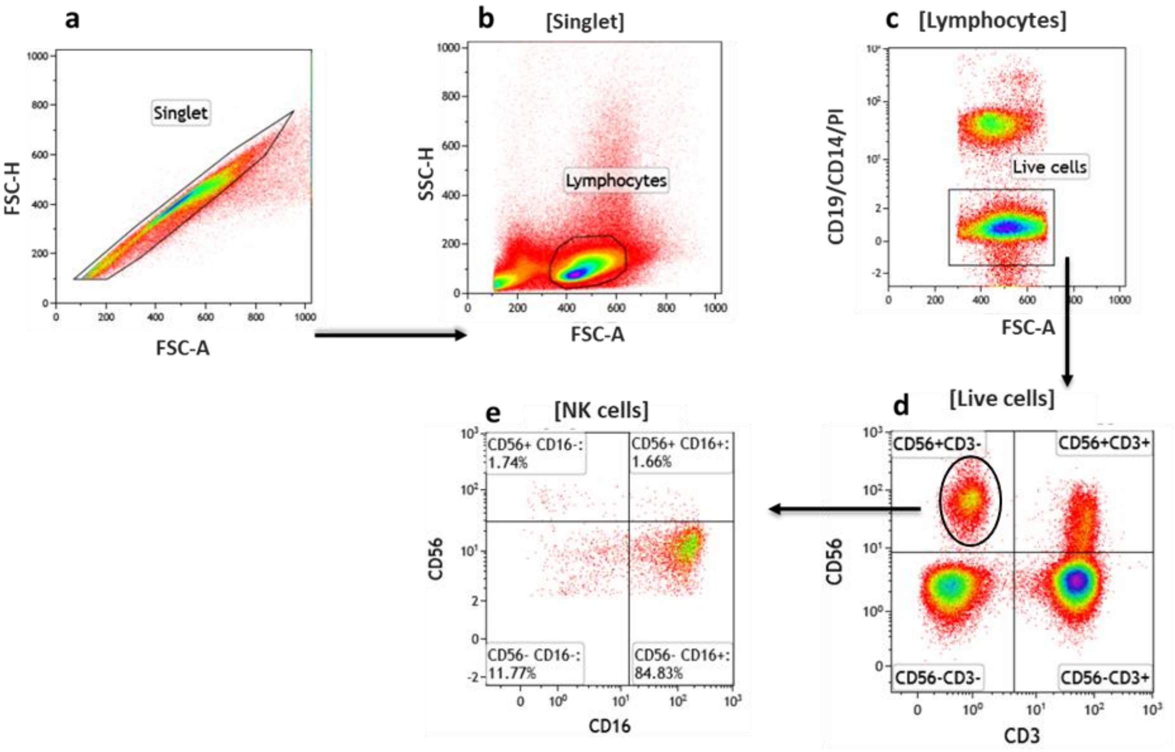

Supplementary Figure 1: PBMC were first gated on singlet to remove doublet cells (a), lymphocytes were identified using FSC-A and SSC-H properties (b). Before gating on NK cells, live cells were identified as being PI negative cells, whilst dead cells (PI positive cells), B cells (including tumor B cells) and monocytes were excluded on dump channel (c). NK cells were identified from live population as CD56pos CD3neg PI- (d) and further classified into four different subsets according to the level of CD56 and CD16 expression

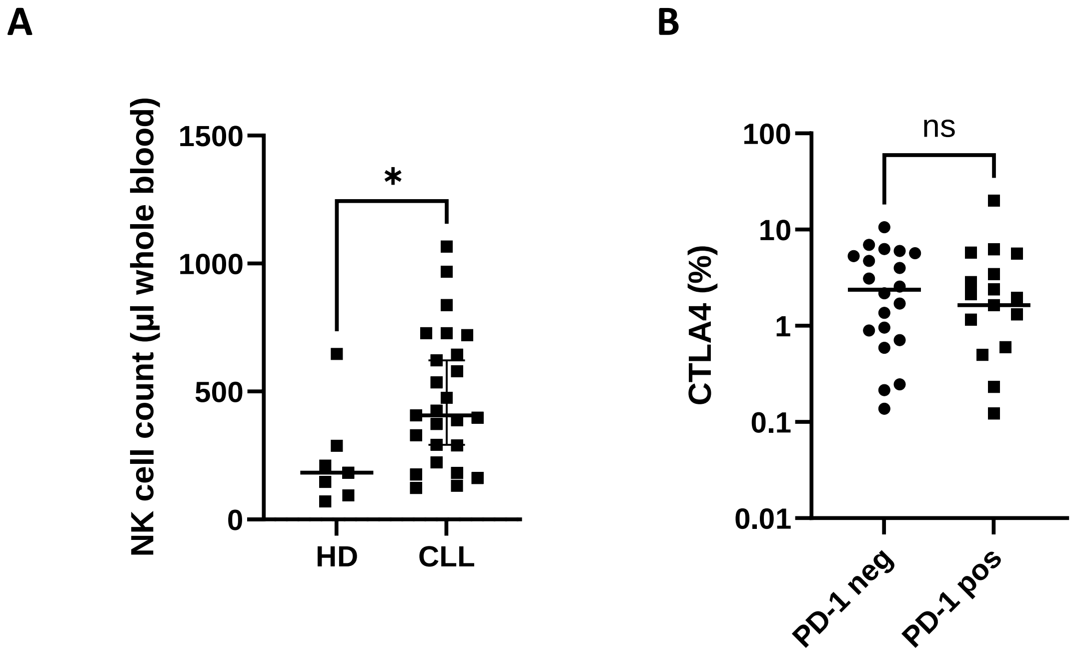

Supplementary Figure 2: NK cell count is increased in patients with CLL

(A) NK cell counts in patients and age-matched healthy donors (HD).

(B) Expression of CTLA-4 on PD-1-positive and PD-1-negative NK cells from patients with CLL. Data shown as percentage of CTLA4-positive cells. Statistical analysis with Mann-Whitney non-parametric test (*p<0.05).

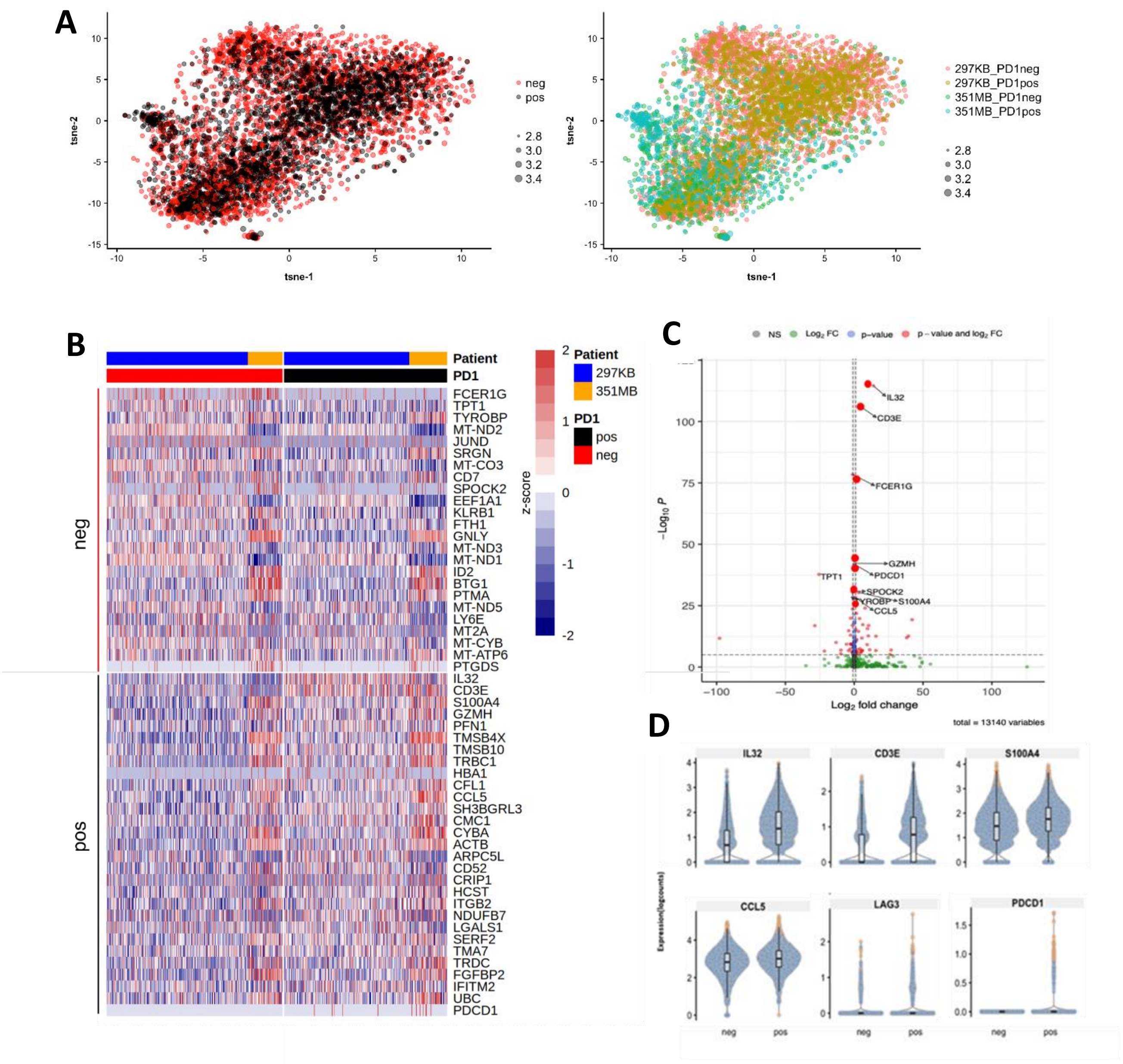

Supplementary Figure 3: Differentiated transcription profile of PD-1^{neg} and PD-1^{pos} NK cells. (A) tSNE embedding of NK cell transcriptomes overlaid with PD1 status and patient labels. Point size represents log10 of the feature (gene) count. (B) Transcriptional signature of PD1^{pos} and PD1^{neg} NK cells showing differentially expressed genes (FDR < 0.01 and absolute logFC > 0.1). (C) Volcano plot summarising the results of PD1^{pos} vs PD1^{neg} differential expression analysis. Point colour represents passing of p-value and fold change thresholds for differential expression. X axis is the Log2 fold change of gene expression level and Y axis is the P value.(D) Violin plots to compare the transcription level of IL-32, CD3E, S100A4, CCL5, LAG3 and PDCD1 genes from the top modulated genes list between PD-1^{pos} versus PD-1^{neg} NK cells, Y axis is normalized log gene expression.

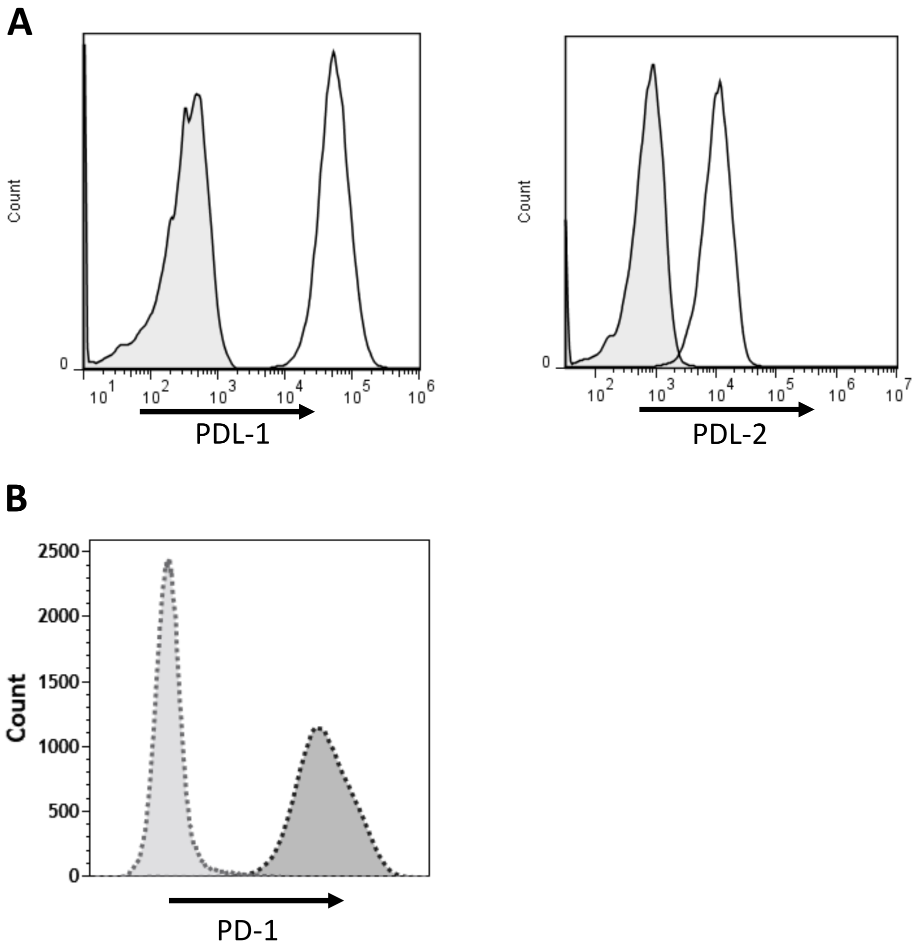

Supplementary Figure 4: (A)**:** Histogram plots to illustrate PDL-1(left panel) and PDL-2 (right panel) expression on 721.221 cell lines, with the filled grey plot as isotype control staining. (B) Histogram plots to illustrate PD-1 expression on PD-1 transduced NK-92 cell lines (Dark grey) compared to wild type control NK cell lines (light grey).
